# Inhibiting the expression of spindle appendix cooled coil protein 1 can suppress tumor cell growth and metastasis and is associated with cancer immune cells in esophageal squamous cell carcinoma

**DOI:** 10.1371/journal.pone.0302312

**Published:** 2024-08-28

**Authors:** Tao Liu, Juan Xu, Qun-Xian Zhang, Yan-Jiao Huang, Wei Wang, Zhu Fu

**Affiliations:** 1 Department of Cardiothoracic Surgery, Taihe Hospital, Hubei University of Medicine, Shiyan City, China; 2 Operating Room, Taihe Hospital, Hubei University of Medicine, Shiyan City, China; 3 Department of Pediatrics, Taihe Hospital, Hubei University of Medicine, Shiyan City, China; UT Austin: The University of Texas at Austin, UNITED STATES

## Abstract

Inhibiting the expression of spindle appendix cooled coil protein 1 (SPDL1) can slow down disease progression and is related to poor prognosis in patients with esophageal cancer. However, the specific roles and molecular mechanisms of SPDL1 in esophageal squamous cell carcinoma (ESCC) have not been explored yet. The current study aimed to investigate the expression levels of SPDL1 in ESCC via transcriptome analysis using data from The Cancer Genome Atlas (TCGA) and Gene Expression Omnibus databases. Moreover, the biological roles, molecular mechanisms, and protein networks involved in SPDL1 were identified using machine learning and bioinformatics. The cell counting kit-8 assay, EdU staining, and transwell assay were used to investigate the effects of inhibiting SPDL1 expression on ESCC cell proliferation, migration, and invasion. Finally, the correlation between the SPDL1 expression and cancer immune infiltrating cells was evaluated by analyzing data from the TCGA database. Results showed that SPDL1 was overexpressed in the ESCC tissues. The SPDL1 expression was related to age in patients with ESCC. The SPDL1 co-expressed genes included those involved in cell division, cell cycle, DNA repair and replication, cell aging, and other processes. The high-risk scores of SPDL1-related long non-coding RNAs were significantly correlated with overall survival and cancer progression in patients with ESCC (P < 0.05). Inhibiting the SPDL1 expression was effective in suppressing the proliferation, migration, and invasion of ESCC TE-1 cells (P < 0.05). The overexpression of SPDL1 was positively correlated with the levels of Th2 and T-helper cells, and was negatively correlated with the levels of plasmacytoid dendritic cells and mast cells. In conclusion, SPDL1 was overexpressed in ESCC and was associated with immune cells. Further, inhibiting the SPDL1 expression could effectively slow down cancer cell growth and migration. SPDL1 is a promising biomarker for treating patients with ESCC.

## Introduction and preliminaries

Esophageal squamous cell carcinoma (ESCC) is a subtype of esophageal cancer (ESCA) that is common globally and domestically [1]. In the past few decades, comprehensive treatment with surgical methods has been the primary approach for treating patients with early-stage ESCC. However, the prognosis of patients with advanced- and intermediate-stage ESCC is often extremely poor due to loss of surgical options. In recent years, preoperative adjuvant chemotherapy combined with immunotherapy has been effective in downstaging in patients with this type of cancer. Hence, the prognosis of patients with ESCC undergoing surgery has improved [2,3]. Yang et al., reported that patients with ESCA treated with chemotherapy combined with immunotherapy did not present with significant toxic effects or perioperative mortality. In addition, patients with ESCA commonly responded well to chemotherapy combined with immunotherapy [3]. Despite these promising developments, the prognosis of patients with ESCC remain unsatisfactory.

Recently, the spindle apparatus coiled coil protein 1 (SPDL1) gene has gained increasing attention. Previous studies have revealed that SPDL1 was associated with digestive tract malignancies and tumor progression [4–7]. For example, decreased SPDL1 expression was related to poor prognosis in patients with colorectal cancer (CRC). Moreover, it was considered an independent risk factor of survival time in patients with CRC [5]. Myocardin-related transcription factor B, a tumor suppressor gene in CRC progression, inhibits cell invasion and migration by suppressing the expression of melanoma cell adhesion molecule and SPDL1. Decreased SPDL1 expression in CRC cells significantly increased tumor development, and it was found to be associated with poor prognosis in patients with cancer [7]. Based on a recent study, the expression level of SPDL1 decreased significantly in ESCA, and a lower SPDL1 expression was associated with worse prognosis in patients with ESCA [6]. Moreover, suppressing SPDL1 expression slows down the ESCA progression [6]. However, the different subtypes of ESCA, such as esophageal adenocarcinoma and ESCC, have various prognoses, and the correlation between SPDL1 expression and ESCC progression has not been completely understood. Hence, this study aimed to identify the SPDL1 levels based on the ESCC data from The Cancer Genome Atlas (TCGA) and Gene Expression Omnibus (GEO) databases, and the functions, mechanisms, and protein networks involved in SPDL1 using bioinformatics and cell experiments. In addition, the correlation between the SPDL1 levels and the immune cells in ESCC was examined to identify a novel candidate molecule in ESCC treatment.

## Methods

### Data on patients with ESCC from the TCGA and GEO databases

The TCGA database was utilized to obtain transcriptome data on cancer and normal tissues collected from patients with ESCC. In particular, the data on cancer and normal esophageal tissues, in the TPM data type, were downloaded from the TCGA website. Furthermore, data on the clinical characteristics of patients with ESCC, including T stage, N stage, M stage, pathological stage, sex, age, and grade, were obtained from the TCGA database. In addition, expression data on the cancer and normal tissues of patients with ESCC were downloaded from the GEO database. The datasets included were GSE20347, GSE23400, GSE29001, GSE45670, and GSE161533.

### Correlation between the SPDL1 expression and clinical features in ESCC

The SPDL1 expression levels in normal and ESCC tissues were analyzed via expression analysis using the TCGA and GEO databases. And the data on the SPDL1 expression in the cancer tissues of patients with ESCC in the TCGA database were merged with the data on the clinical characteristics of patients with ESCC. The correlation between the SPDL1 expression and the clinical characteristics of patients with ESCC was assessed by categorizing them into the SPDL1 overexpression and suppression SPDL1 suppression groups according to the median SPDL1 expression.

### Co-expressed genes and long non-coding RNAs of SPDL1

Pearson correlation analysis was conducted to determine the correlation between genes and long non-coding RNAs (lncRNAs) [8,9]. The SPDL1 co-expressed genes and lncRNAs were selected via filtering based on a correlation coefficient (r), with an absolute value > 0.6, which was classified as strongly correlated. Furthermore, the correlation between these genes and lncRNAs was illustrated using scatter and network plots.

### Biological functions, mechanisms, and protein network involved in SPDL1

The online David database was utilized to analyze the biological functions and potential signaling mechanisms of multiple genes. After inputting the SPDL1 co-expressed genes into the David database, the biological functions and potential signaling mechanisms were analyzed. Next, a filter condition (P-value < 0.05) was used to refine the results. Furthermore, the TISDIB database, an immune-related database, was used to explore the functions and mechanisms related to the SPDL1 gene. In addition, a protein network of the SPDL1 co-expressed genes was constructed using the STRING database and visualized with the Cytoscape software.

### Correlation between the risk models and prognosis in patients with ESCC

The survival data of patients with ESCC were obtained from the TCGA database and merged with the data on the expression of SDPL1, AC091057.1, AC107214.1, AC004943.2, and AC012073.1. LASSO regression analysis was conducted to explore the correlation between the expression levels of these factors and the overall survival (OS) and progression-free interval (PFI) in patients with ESCC. The risk models were constructed based on the LASSO results. In addition, survival analysis was performed to identify the correlation between a high-risk score and the OS and PFI in patients with ESCC.

### Suppression of the SPDL1 expression in the TE-1 cell model

In this study, ESCC TE-1 cells were cultured in the RMPI-1640 medium supplemented with 10% fetal bovine serum (FBS) and 1% penicillin and streptomycin. The cell model of the suppressed SPDL1 expression was constructed via the siRNA interference in TE-1 cells during the growth stage, as confirmed on reverse transcription-polymerase chain reaction (RT-PCR) and Western blot analysis.

### Identification of the cell model of the suppressed SPDL1 expression via western blot analysis and RT-PCR

After siRNA transfection in TE-1 cells, the total proteins and RNAs were collected from the control group (NC) and the group with suppressed SPDL1 expression (si-SPDL1) using RNA and protein lysates, respectively. GGGAGAAGUUUAUCGAUUATT is the siRNA sequenced SPDL1. The total RNA and protein solutions collected from these groups were quantified, and the PCR amplification and antibody incubation were performed according to the standard RT-PCR and western blot protocols [10]. The SPDL1 antibody was purchased from Proteintech (No. 24689-1-AP). Finally, the relative expression levels of SPDL1 in the NC and si-SPDL1 cell groups were calculated.

### CCK-8

TE-1 cells were transfected to achieve good growth status and then divided into the NC and si-SPDL1 groups. After plating 3,000 cells in a 96-well plate, 10 μl of the CCK-8 solution was added to both groups during TE-1 cell attachment. After 1 h, the optical density of the two groups was measured using a microplate reader and recorded as 0 h. Subsequently, 10 μl of the CCK-8 solution was added to the NC and si-SPDL1 groups at 24, 48, and 72 h. Next, the cell optical density was measured with a microplate reader and plotted on a line chart.

### EdU staining

In this study, the effects of SPDL1 on TE-1 cell proliferation was examined via EdU staining. After ESCC TE-1 cell transfection and growth to the appropriate stage, a cell section was prepared and stained, then protected from light, and incubated at room temperature. Next, methanol fixation, DNA staining, and immediate observation after PBS washing were performed to determine TE-1 cell proliferation in the NC and si-SPDL1 groups.

### Transwell assay

Standard methods were followed to prepare the Transwell chambers with or without Matrigel [10]. TE-1 cells with good growth condition after cell transfection were digested and adjusted to a density of 2×10^5^ cells for each of the NC and si-SPDL1 groups. A 200 μl cell suspension was then added to the upper chamber of the Transwell chamber. Then, 800 μl of the RPMI-1640 medium containing FBS was added to the lower chamber. Precautions were taken to prevent creating bubbles. Photographic images and relevant statistical data were collected for 24 h after routine incubation.

### Association between the SPDL1 expression and immune cell infiltration in ESCC

First, the relative levels of immune cells in the TCGA ESCC tissues were calculated using the ssGSEA method [10]. Next, the SPDL1 expression data were then extracted from the TCGA ESCC tissues. To investigate the correlation between the decreased SPDL1 expression and immune cell levels via Pearson correlation analysis, immune cell levels were combined with the SPDL1 expression data using the Perl language. In addition, the immune cell levels were analyzed for statistical significance in the high- and low-expression SPDL1 groups. Overall, this analysis aimed to better understand the correlation between the SPDL1 expression and immune cell infiltration in ESCC.

### Statistical analysis

To further investigate the significance of SPDL1 levels in ESCC, the *t*-test was utilized. The t-test aimed to explore the association between the SPDL1 levels and TE-1 cell proliferation, migration, and invasion. In addition, Pearson correlation analysis was conducted to better understand the co-expressed genes and lncRNAs in relation to the SPDL1 expression. Further, the correlation between the SPDL1 expression and immune cells was also explored using this analysis technique. A P value of < 0.05 was considered statistically significant, and it ensures statistical rigor in the analysis.

## Results

### SPDL1 was overexpressed in ESCC

**Fig 1:**
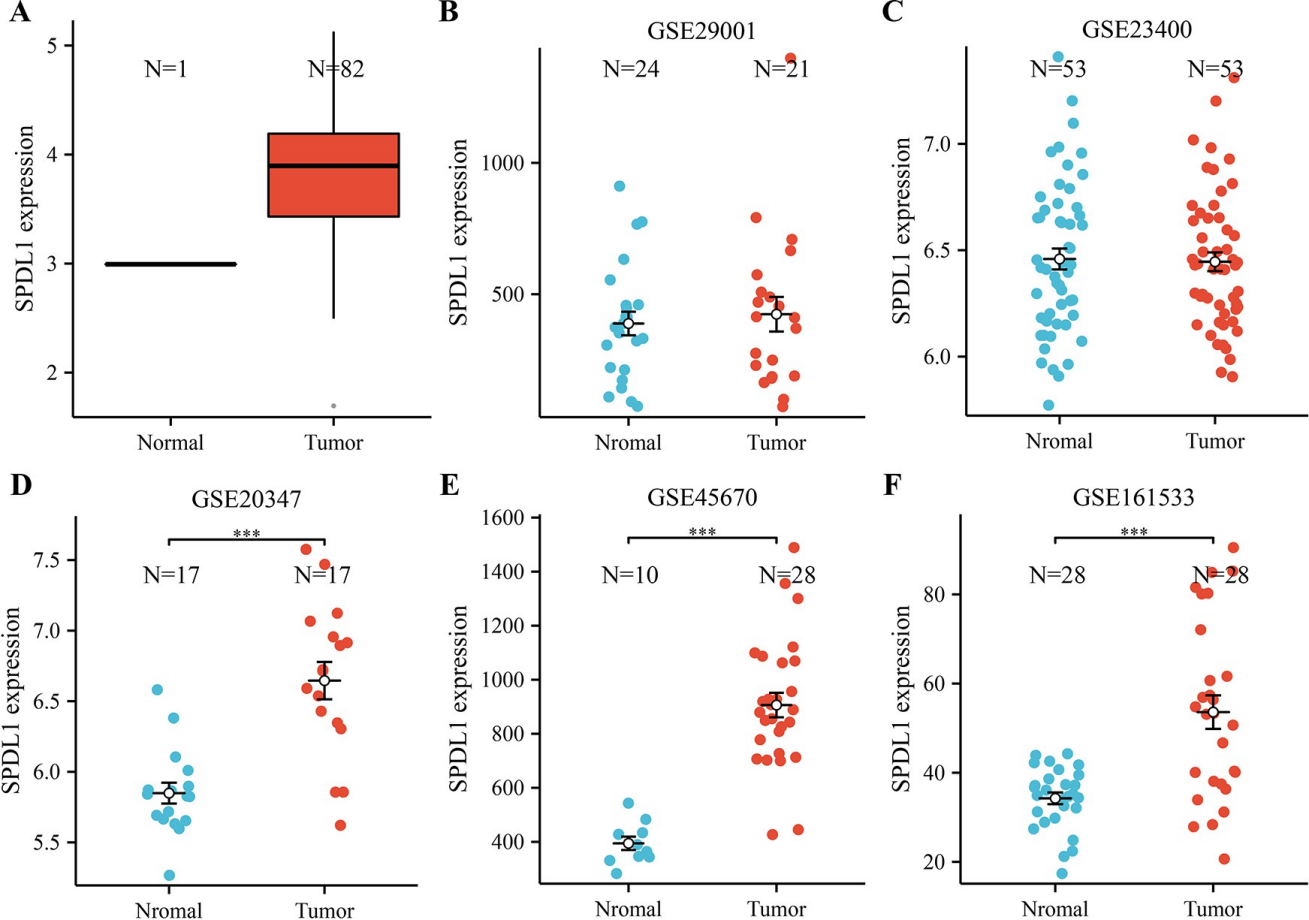
Decreased SPDL1 expression in the ESCC tissues. (A) TCGA database (B–F) Datasets from the GEO database. Note: TCGA, The Cancer Genome Atlas; GEO, Gene Expression Omnibus; ESCC, esophageal squamous cell carcinoma.

Based on the data from the TCGA database, the expression of SPDL1 in the ESCC tissues was significantly higher than that in the normal tissues (Fig 1A). The GSE29001, GSE23400, GSE20347, GSE45670, and GSE161533 datasets from the GEO database had similar results. The SPDL1 expression in ESCC tissues was significantly enhanced (Fig 1B–1F). Further, the expression levels of SPDL1 in the normal and ESCC tissues were statistically significant in the GSE20347, GSE45670, and GSE161533 datasets (P < 0.05). Table 1 shows the association between the SPDL1 expression and clinical features in ESCC. In particular, the expression of SPDL1 was significantly correlated with age, but not with T, N, and M stages and sex, in patients with ESCC.

**Table 1:** The correlation between SPDL1 expression and clinical features in ESCC.

Characteristics	Low expression of SPDL1	High expression of SPDL1	P value
N	41	41	
Pathologic T stage			0.735294151
T1	5 (6.3%)	3 (3.8%)	
T2	15 (19%)	12 (15.2%)	
T3	19 (24.1%)	22 (27.8%)	
T4	2 (2.5%)	1 (1.3%)	
Pathologic N stage			0.145683929
N0	27 (34.6%)	19 (24.4%)	
N1	10 (12.8%)	16 (20.5%)	
N2	4 (5.1%)	1 (1.3%)	
N3	0 (0%)	1 (1.3%)	
Pathologic M stage			0.980667479
M0	36 (49.3%)	34 (46.6%)	
M1	1 (1.4%)	2 (2.7%)	
Pathologic stage			0.597438255
Stage I	5 (6.3%)	2 (2.5%)	
Stage II	25 (31.6%)	22 (27.8%)	
Stage III	10 (12.7%)	12 (15.2%)	
Stage IV	1 (1.3%)	2 (2.5%)	
Gender			0.211386544
Female	8 (9.8%)	4 (4.9%)	
Male	33 (40.2%)	37 (45.1%)	
Age			0.011061361
< = 60	21 (25.6%)	32 (39%)	
> 60	20 (24.4%)	9 (11%)	

### Biological functions involved in the SPDL1 co-expressed genes

**Fig 2:**
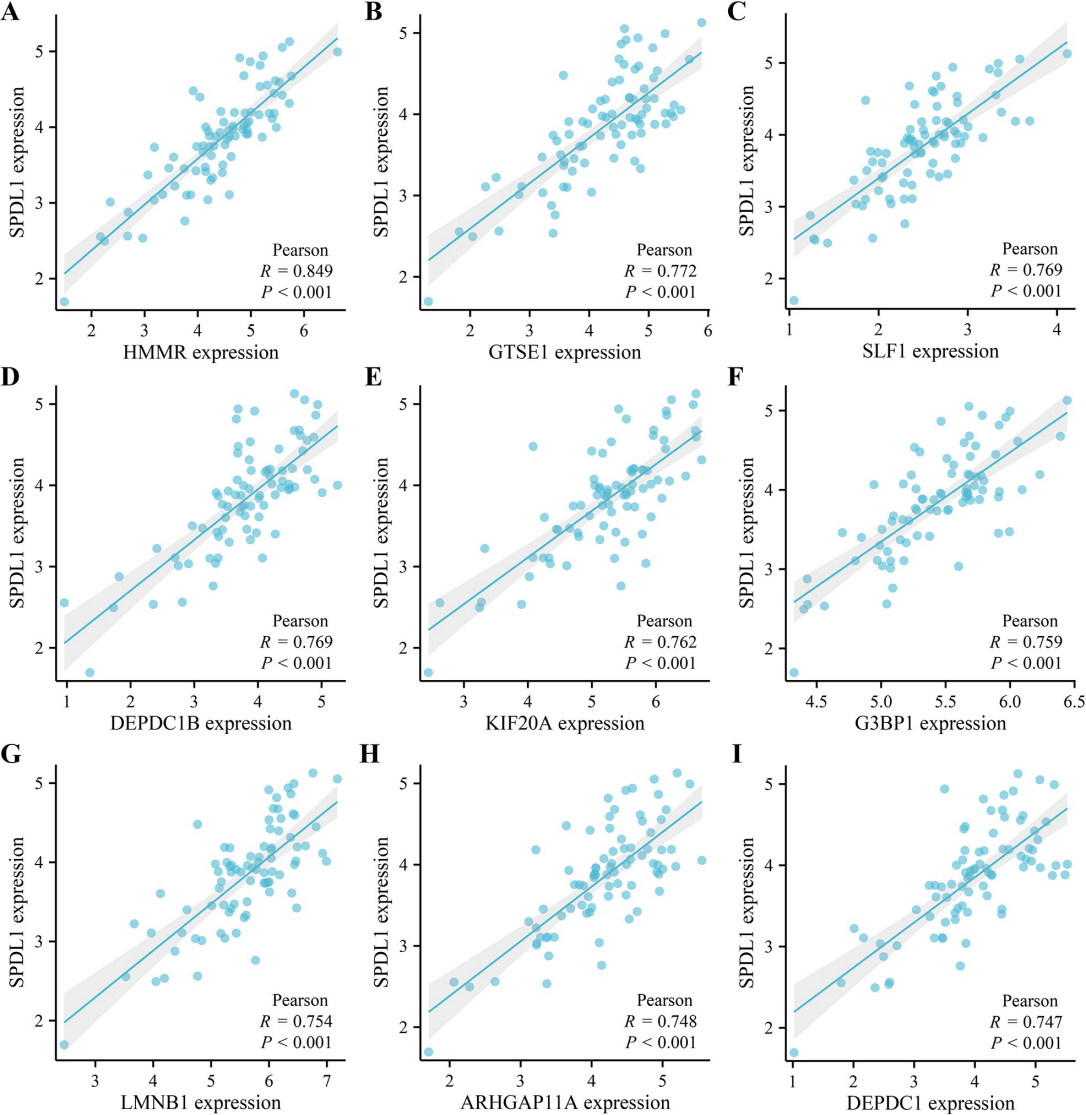
Top nine SPDL1 co-expressed genes in the ESCC tissues. (A) HMMR (B) GTSE1 (C) SLF1 (D) DEPDC1B (E) KIF20A (F) G3BP1 (G) LMNB1 (H) ARHGAP11A (I) DEPDC1. Note: ESCC, esophageal squamous cell carcinoma.

**Fig 3:**
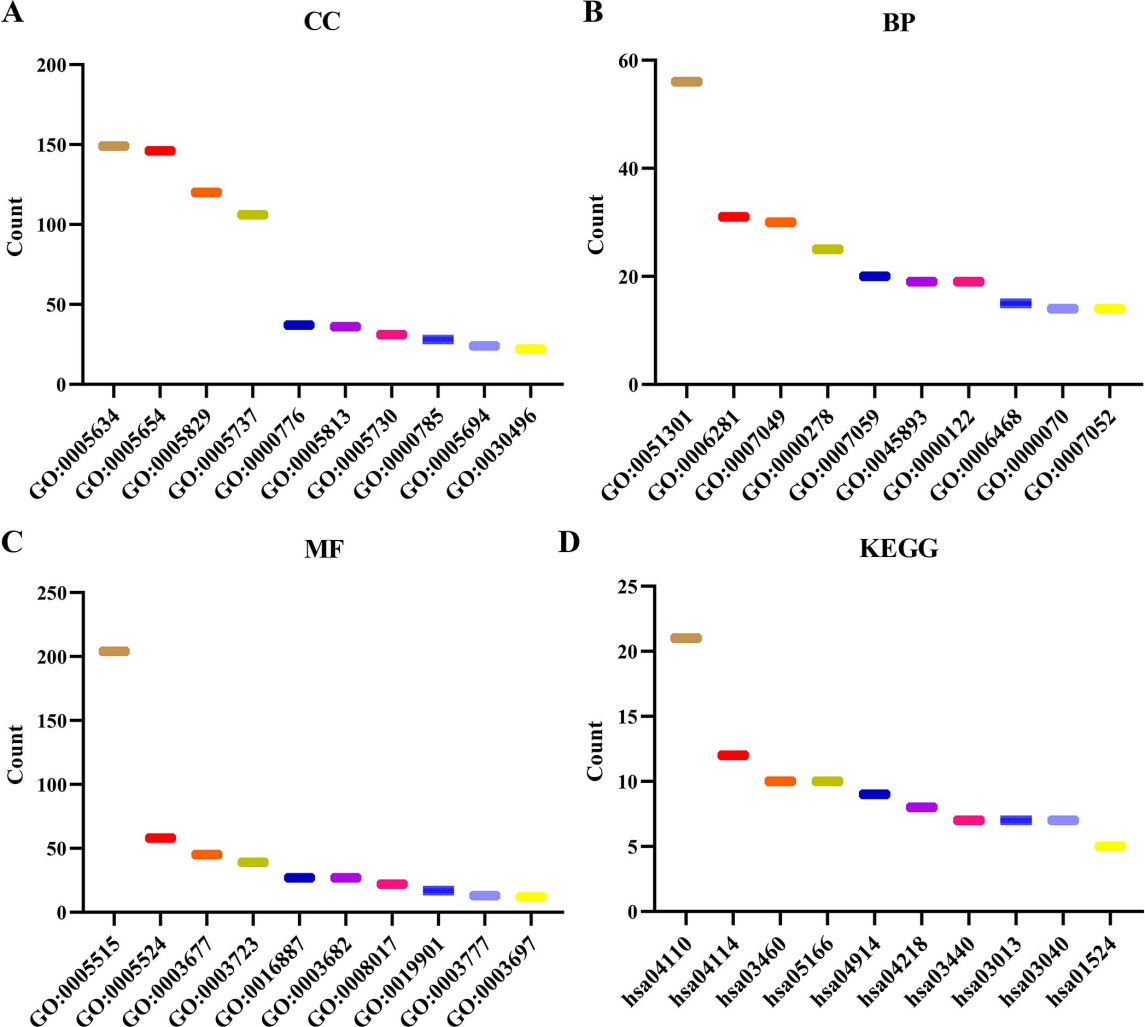
The roles and pathways of the SPDL1 co-expressed genes. (A) CC (B) BP (C) MF (D) KEGG. Note: MF, molecular function; CC, cellular component; BP, biological process; KEGG, Kyoto Encyclopedia of Genes and Genomes.

This analysis revealed 247 co-expressed genes for SPDL1. Moreover, nine SPDL1 co-expressed genes (HMMR, GTSE1, SLF1, DEPDC1B, KIF20A, G3BP1, LMNB1, ARHGAP11A, and DEPDC1) were showed (Fig 2). These genes were involved in numerous biological processes, including cell division, chromosome separation, DNA repair, cell cycle regulation, ATP binding, ATPase activity, microtubule binding, and other important cellular functions, as shown via an analysis in the David database (Fig 3A–3C). In the TISDIB database, the SPDL1 gene was involved in the regulation of mitotic sisters, chromatid separation, cell cycle checkpoints, establishment of mitotic spindle orientation, chromosome separation, mitotic mitosis, nuclear chromosome separation, and other vital cellular processes (Table 2).

**Table 2:** The roles of SPDL1 in the TISIDB database.

GO type	Catalog	
BP	GO:0000070	Mitotic sister chromatid segregation
	GO:0000075	Cell cycle checkpoint
	GO:0000132	Establishment of mitotic spindle orientation
	GO:0000226	microtubule cytoskeleton organization
	GO:0000819	sister chromatid segregation
	GO:0007059	Chromosome segregation
	GO:0007062	Sister chromatid cohesion
	GO:0007067	Mitotic nuclear division
	GO:0007080	Mitotic metaphase plate congression
	GO:0007163	Establishment or maintenance of cell polarity
	GO:0030010	Establishment of cell polarity
	GO:0031577	Spindle checkpoint
	GO:0034501	Protein localization to kinetochore
	GO:0034502	Protein localization to chromosome
	GO:0040001	Establishment of mitotic spindle localization
	GO:0050000	Chromosome localization
	GO:0051293	Establishment of spindle localization
	GO:0051294	Establishment of spindle orientation
	GO:0051303	Establishment of chromosome localization
	GO:0051310	Metaphase plate congression
	GO:0051640	Organelle localization
	GO:0051653	Spindle localization
	GO:0051656	Establishment of organelle localization
	GO:0071459	Protein localization to chromosome, centromeric region
	GO:0098813	Nuclear chromosome segregation
MF	GO:0043515	Kinetochore binding
CC	GO:0000775	Chromosome, centromeric region
	GO:0000776	Kinetochore
	GO:0000777	Condensed chromosome kinetochore
	GO:0000779	Condensed chromosome, centromeric region
	GO:0000793	Condensed chromosome
	GO:0000922	Spindle pole
	GO:0000940	Condensed chromosome outer kinetochore
	GO:0005819	Spindle
	GO:0098687	Chromosomal region

Note: BP, biological process; CC, cellular component; MF, Molecular function.

### The mechanisms and protein network involved in the SPDL1 co-expressed genes

**Fig 4:**
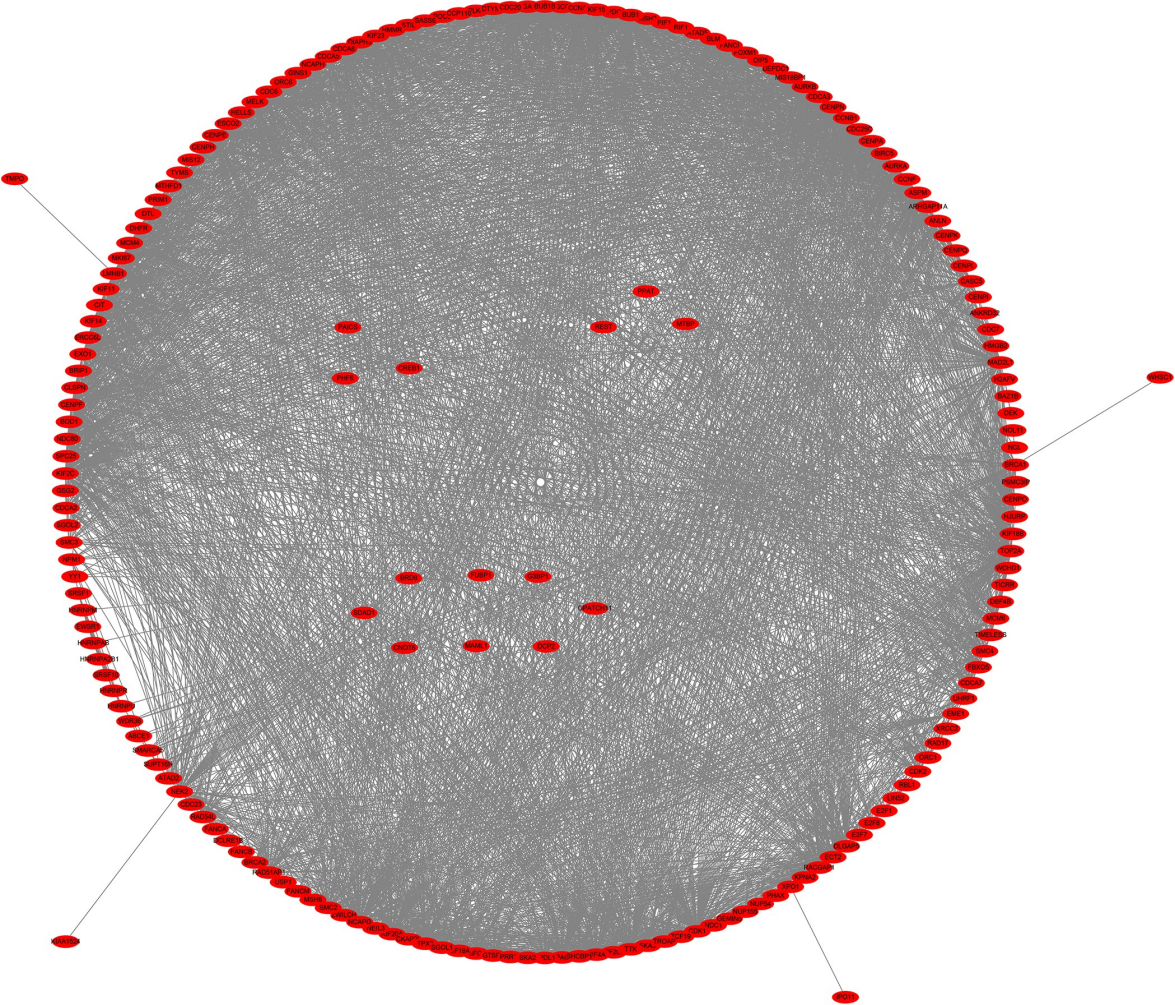
The PPI network of the SPDL1 co-expressed genes.

Based on the data in the David database, the SPDL1 co-expressed genes were involved in several mechanisms that are important in the pathogenesis of ESCC. These mechanisms include the regulation of cell cycle, oocyte meiosis, homologous recombination, nuclear cytoplasmic transport, cell aging, spliceosome, platinum drug resistance, mismatch repair, and others (Fig 3D and Table 3). Furthermore, the protein–protein interaction (PPI) network was performed to better understand the correlation between the SPDL1 co-expressed genes and related genes. Fig 4 shows the PPI network in detail and the interactions between the identified genes, including SPDL1.

**Table 3:** The pathways of the SPDL1co-expressed genes.

Term	Count	P value
hsa04110: Cell cycle	21	8.35E-18
hsa03460: Fanconi anemia pathway	10	8.82E-09
hsa04114: Oocyte meiosis	12	2.82E-07
hsa03440: Homologous recombination	7	6.66E-06
hsa04914: Progesterone-mediated oocyte maturation	9	2.16E-05
hsa05166: Human T-cell leukemia virus 1 infection	10	0.001030983
hsa03013: Nucleocytoplasmic transport	7	0.001522889
hsa04218: Cellular senescence	8	0.00214048
hsa03040: Spliceosome	7	0.007021874
hsa01524: Platinum drug resistance	5	0.009986068
hsa00670: One carbon pool by folate	3	0.022010614
hsa03430: Mismatch repair	3	0.028660224

### Significant correlation between the risk model of the SPDL1 co-expressed lncRNAs and poor prognosis in patients with ESCC

**Fig 5:**
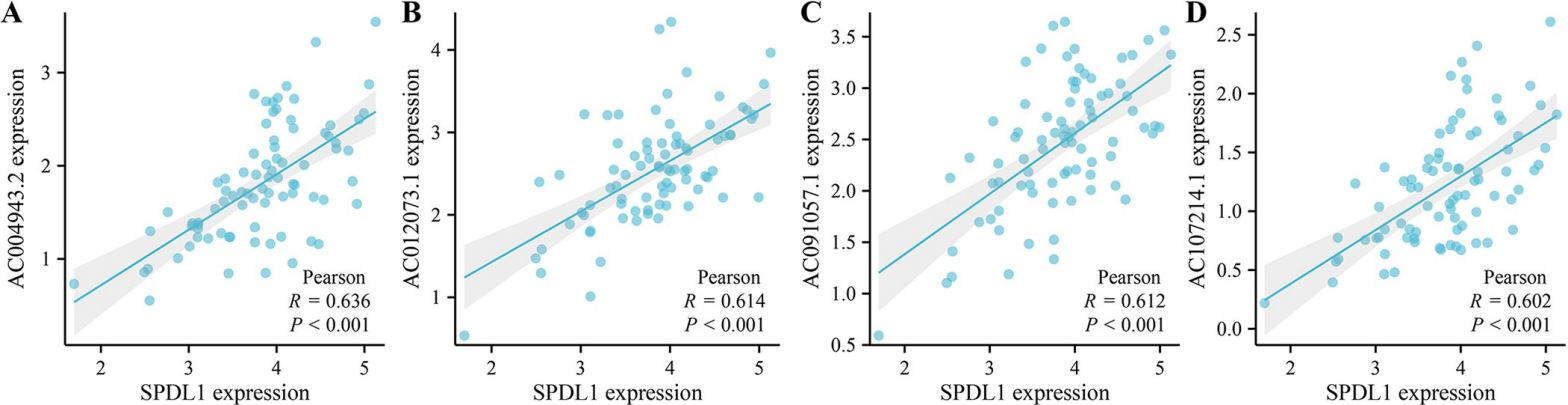
SPDL1 co-expressed lncRNAs. (A) AC004943.2. (B) AC012073.1. (C) AC091057.1. (D) AC107214.1.

**Fig 6:**
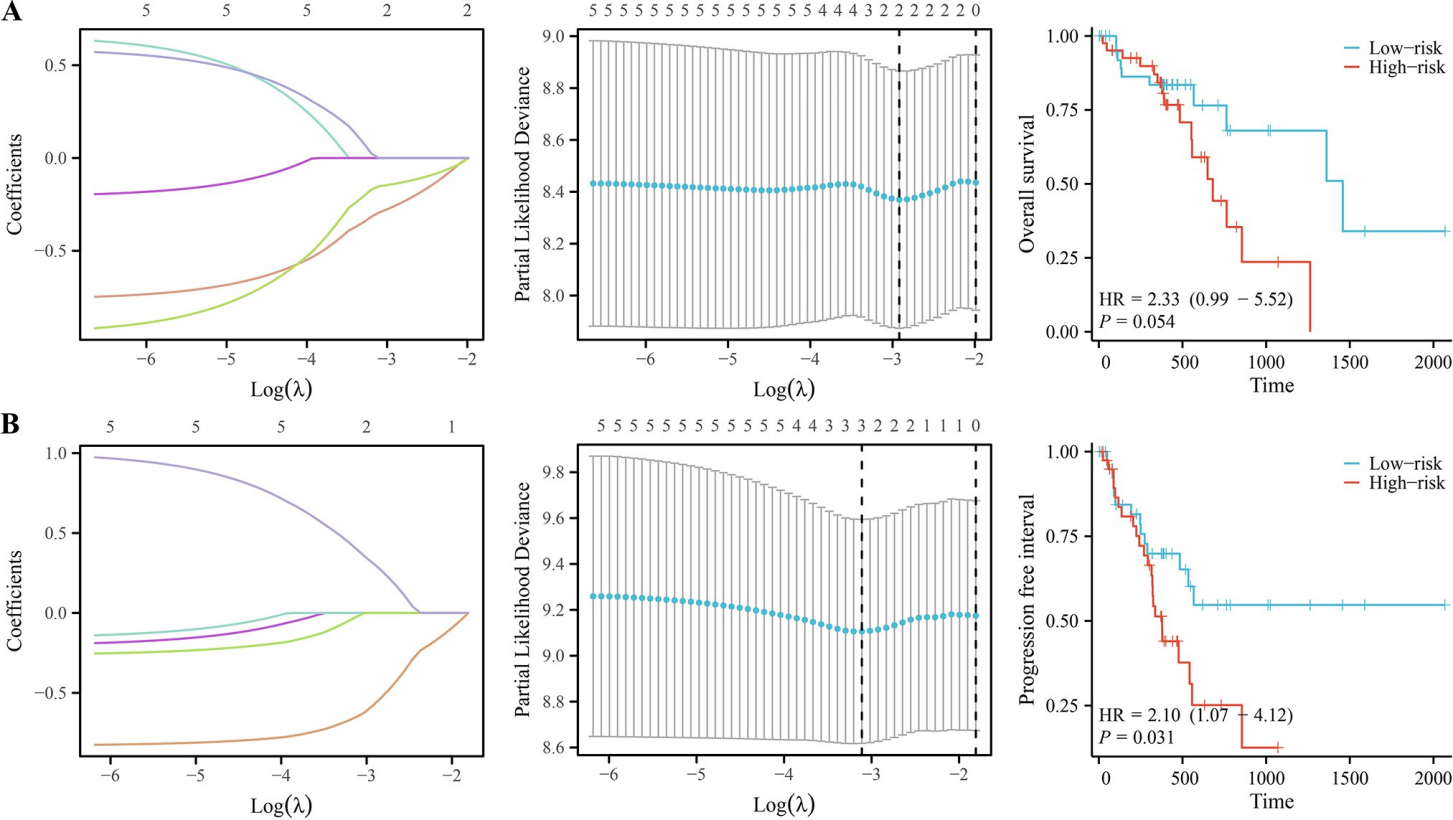
SPDL1 co-expressed lncRNAs were related to the dismal prognosis in ESCC. (A) OS. (B) PFI.

The correlation analysis indicated a positive correlation between the SPDL1 expression levels and the expression levels of lncRNAs AC091057.1, AC107214.1, AC004943.2, and AC012073.1 (Fig 5). A LASSO regression analysis was then conducted to further explore the correlation between the expression levels of these lncRNAs and SPDL1 and prognosis in patients with ESCC. Results showed that the expression of AC004943.2 and AC012073.1 was a significant risk factor of OS in patients with ESCC. Patients with high-risk ESCC in the risk model constructed based on AC004943.2 and AC012073.1 had a significantly shorter OS (Fig 6A). In addition, AC004943.2, AC012073.1, and SPDL1 were also considered as risk factors for PFI in patients with ESCC, and those in the high-risk group had a shorter PFI (Fig 6B).

### Significant suppression of the proliferation, migration, and invasion of ESCC cells via the inhibition of the SPDL1 expression

**Fig 7:**
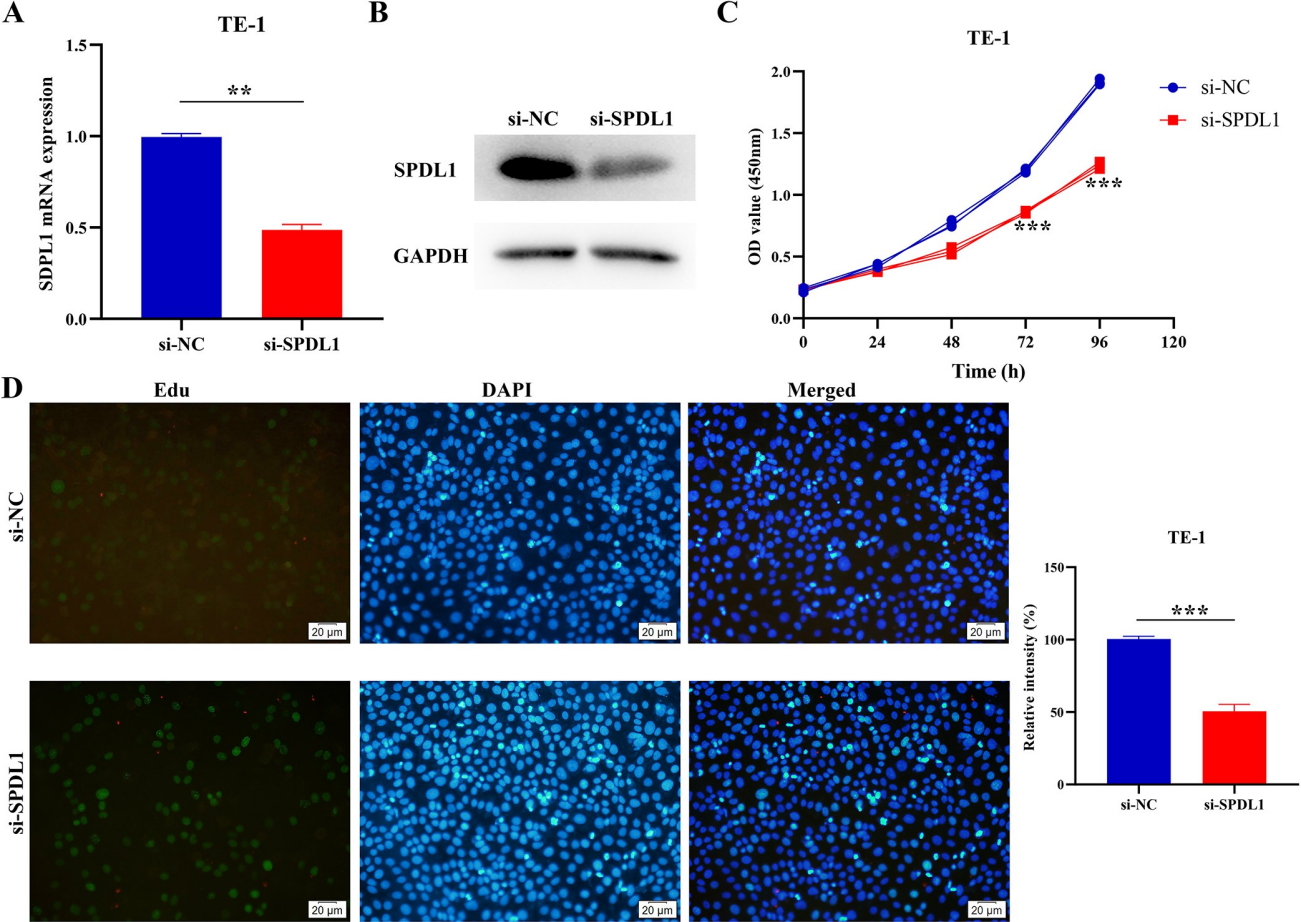
Inhibiting SPDL1 expression could suppress ESCC cell proliferation. (A) Cell model using PCR (B) Cell model using Western blot analysis (C) Cell proliferation using the CCK-8 assay (D) Cell proliferation using EdU staining.

**Fig 8:**
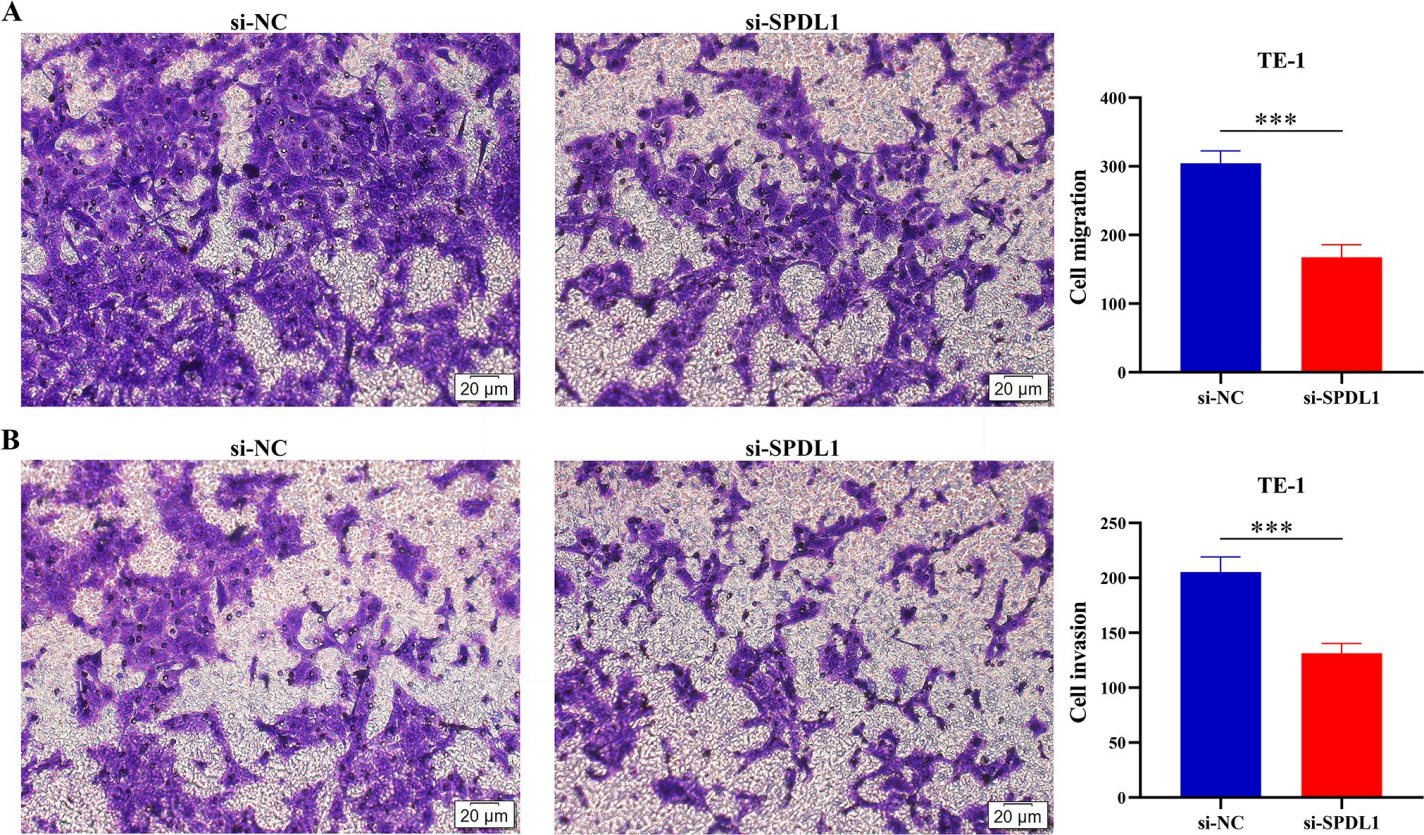
Inhibiting SPDL1 expression could suppress ESCC cell migration and invasion. (A) Cell migration. (B) Cell invasion.

A cell model for inhibiting SPDL1 expression in TE-1 cells was successfully developed using RT-PCR and western blot techniques (Fig 7A and 7B). Results from the CCK-8 assay revealed that inhibiting SPDL1 expression resulted in a significant decrease in cell proliferation at 72 and 96 h (Fig 7C). These results were consistent with those of the EdU assay, which showed that inhibiting SPDL1 expression could significantly suppress TE-1 cell proliferation (Fig 7D). Therefore, the average number of cells in the control group was approximately twice that of the expression inhibition group (Fig 7D). In addition, transwell experiments revealed that inhibiting the SPDL1 expression could suppress the migration and invasion of TE-1 cells (Fig 8).

### Correlation between the expression of SPDL1 and immune cell infiltration in ESCC

**Fig 9:**
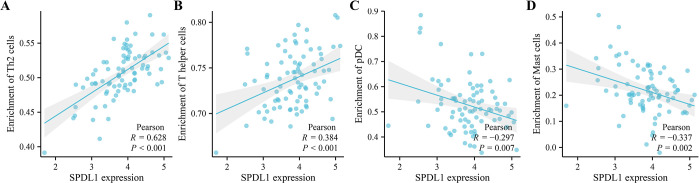
SPDL1 was related to the immune cells in ESCC. (A) Th2 cells (B) T-helper cells (C) pDC (D) Mast cells.

**Fig 10:**
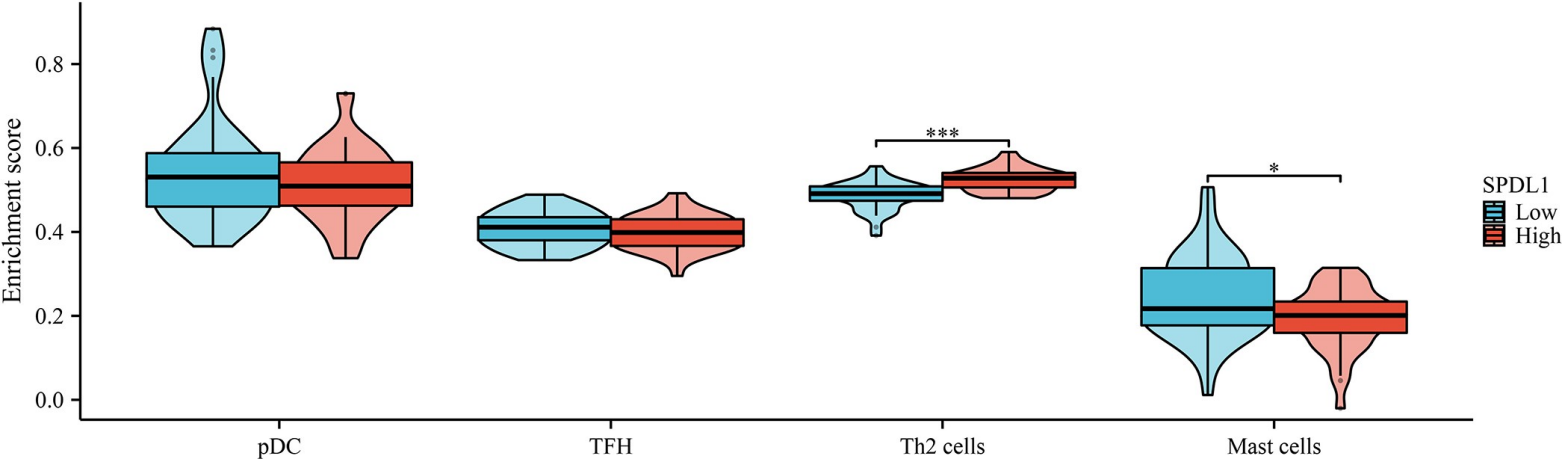
The levels of immune cells in the high- and low-SPDL1 expression groups.

Pearson correlation analysis revealed that the overexpression of SPDL1 was positively associated with the levels of Th2 and T-helper cells (Fig 9A and 9B). Conversely, the SPDL1 expression was negatively correlated with the levels of plasmacytoid dendritic cells and mast cells (Fig 9C and 9D). Furthermore, statistical analysis indicated that the levels of Th2 cells and mast cells significantly differed between the high- and low-SPDL1 expression groups (Fig 10). Therefore, SPDL1 is involved in regulating immune cell infiltration in ESCC.

## Discussion

Due to the unsatisfactory prognosis of patients with ESCA, researchers explored approaches and techniques that can improve survival rates. Recent studies have revealed a correlation between gene expression changes and cancer progression and prognosis in patients with ESCA [11–14]. For example, an enhanced expression of secreted phosphoprotein 1 (SPP1) could promote DNA damage repair and tumor cell survival in ESCA, thereby leading to resistance to radiotherapy [12]. The overexpression of erythropoietin-producing hepatocyte receptor B3 (EphB3) was found to be significantly associated with lymph node metastasis, differentiation, pathological stage, and survival time in patients with ESCC. The inhibition of EphB3 expression resulted in reduced ESCC cell proliferation, migration, invasion, and tumor growth via the dysregulation of the epithelial-mesenchymal transition process [14]. Recently, Liu et al. reported that SPDL1 was overexpressed in ESCA and was associated with poor prognosis, growth, and metastatic potential [6]. However, the connection between the SPDL1 levels and ESCC has not been comprehensively evaluated yet. The current analysis of data from the TCGA and GEO databases has revealed that SPDL1 was overexpressed in the ESCC tissues. Further examination found that the expression of SPDL1-related lncRNAs AC004943.2 and AC012073.1 were correlated with a shorter OS in patients with ESCC. To develop a risk model, a model based on these factors was developed. Results showed that patients with high-risk ESCC had a significantly shorter OS. In addition, SPDL1 and SPDL1-related lncRNAs AC004943.2 and AC012073.1 were considered as risk factors for PFI in ESCC. Patients with high-risk ESCC in the PFI risk model had a shorter PFI. Research has confirmed that the inhibition of AC012073.1 in the AC012073.1 aggregation nucleus can inhibit colony formation, proliferation, invasion, and migration abilities [15]. Therefore, SPDL1 can play an important role in the development of ESCC.

A modality combining bioinformatics with cellular experiments was frequently used to validate the role of genes in cancer [16,17]. For example, the RAD51-associated protein 1 (RAD51AP1) expression levels were significantly upregulated in the ESCC tissues. Its overexpression was associated with a shorter survival in patients with ESCC. This gene is also positively correlated with Th2 cells and T-helper cells. Inhibiting the expression of RAD51AP1 significantly impedes ESCC cell proliferation, cell cycle arrest, migration, invasion, and promotes cell apoptosis [16]. The current study also revealed that SPDL1 has similar effects. It was involved in essential cellular processes, such as cell division, DNA repair, cell cycle regulation, establishment of mitotic spindle orientation and mitotic sisters, cell cycle checkpoint, chromosome separation, mitotic mitosis, and nuclear chromosome separation. Nevertheless, our TE-1 cell model experiments using the CCK-8 assay, EDU staining, and transwell assay confirmed that the inhibition of SPDL1 expression significantly impaired cancer cell proliferation, migration, and invasion. These findings indicate that suppressing the SPDL1 expression could potentially delay ESCC progression. In addition, the SPDL1 co-expressed genes are related to cancer progression [17–26]. For example, the DEP domain containing 1 (DEPDC1) is overexpressed in gastric adenocarcinoma tissues. The overexpression of DEPDC1 is correlated with metastasis and the differentiation of gastric adenocarcinoma. The upregulation of DEPDC1 promotes cell cycle transition from G1 phase to S phase, and it is associated with an overall poor survival [25]. Hyaluronan-mediated motility receptor (HMMR) is upregulated in prostate cancer and is associated with poor prognosis. In prostate cancer, HMMR promotes proliferation and metastasis via the mTORC2/AKT pathway by inhibiting ubiquitination and increasing AURKA levels *in vitro* and *in vivo* [17]. The correlation between SPDL1 and the SPDL1 co-expressed genes should be validated via PCR and Western blot analysis in the future.

Th2 cells and mast cells played important roles in immune cell activation and regulation [27–30]. RhoA knockout inhibits T cell activation and TH2 cell differentiation *in vitro*, and prevent the development of allergic airway inflammation in vivo. RhoA could regulate the expression of IL-4 receptors and TH2-specific signaling events [27]. Mast cells were innate immune cells in cancer tissues that accumulate in the tumor matrix. Their density levels are related to poor prognosis in patients with cancer, as they regulate tumor cell proliferation, survival, angiogenesis, invasion, and metastasis by interacting with other tumor-infiltrating cells, thereby affecting patient prognosis [30]. In this study, the SDPL1 overexpression was positively correlated with Th2 cell levels, and was negatively correlated with mast cell levels. These correlations were statistically significant for Th2 cells and mast cells in both the high- and low-SDPL1 expression groups, further indicating the significant role of SPDL1 in ESCC progression.

This study showed the biological roles of SPDL1 in ESCC progression by analyzing data from the TCGA and GEO databases and by conducting cell experiments. Our findings suggest that SPDL1 was overexpressed and associated with ESCC progression. However, further validation via PCR and Western blot analysis is required to establish its mechanisms and the association between SPDL1 and the nine SPDL1 co-expressed genes in ESCC cells in the future. Further, SPDL1 was found to be involved in cellular processes such as cell division, DNA repair, DNA replication, cell aging, and others. Therefore, it can be a treatment target in patients with ESCC. In addition, high SPDL1-related lncRNA risk scores were significantly associated with OS and cancer progression in patients with ESCC. Our study also revealed that inhibiting the SPDL1 expression in TE-1 cells decreased proliferation, migration, and invasion. Finally, a positive correlation was observed between the SDPL1 overexpression and Th2 and T-helper cell levels. Conversely, there was a negative correlation between the SDPL1 overexpression and plasmacytoid dendritic and mast cell levels. These findings emphasized the potential of SPDL1 as a novel target for ESCC treatment.

## Conclusion

SPDL1 was overexpressed in ESCC and was associated with immune cells. Inhibiting SPDL1 expression could effectively slow down cancer cell growth and migration. Therefore, SPDL1 can be a promising therapeutic target in ESCC treatment.
